# CT of appendicoliths in adult appendicitis: clinical significance and characteristics of overlooked cases

**DOI:** 10.1007/s00330-023-10273-3

**Published:** 2023-10-14

**Authors:** Rathachai Kaewlai, Pootipong Wongveerasin, Warunyou Lekanamongkol, Dhanawin Wongsaengchan, Wanwarang Teerasamit, Sasima Tongsai, Pramuk Khamman, Anchisa Chatkaewpaisal, Napakadol Noppakunsomboon, Piyaporn Apisarnthanarak

**Affiliations:** 1 Department of Radiology, Faculty of Medicine Siriraj Hospital, Mahidol University, 2 Wanglang Rd, Bangkok Noi, Bangkok, 10700 Thailand; 2 Department of Research, Faculty of Medicine Siriraj Hospital, Mahidol University, 2 Wanglang Rd, Bangkok Noi, Bangkok, 10700 Thailand; 3 Department of Anatomy, Faculty of Medicine Siriraj Hospital, Mahidol University, 2 Wanglang Rd, Bangkok Noi, Bangkok, 10700 Thailand; 4 Department of Surgery, Faculty of Medicine Siriraj Hospital, Mahidol University, 2 Wanglang Rd, Bangkok Noi, Bangkok, 10700 Thailand

**Keywords:** Appendicitis, Adult, Retrospective studies, Tomography (X-ray computed)

## Abstract

**Objectives:**

Accurate computed tomography (CT) identification of appendicoliths in adults with acute appendicitis is crucial as it may preclude nonoperative management due to high risk of failure and complications. This investigation aimed to identify the significance of appendicoliths in acute appendicitis and to evaluate the performance of portovenous-phase (PVP) CT and the consequences of overlooked appendicoliths.

**Methods:**

CT examinations of 324 consecutive patients (mean age 51.9 years, 112 men) with pathologically confirmed acute appendicitis were retrospectively included. Two radiologists independently reviewed the images, and disagreement was resolved by a consensus.

**Results:**

Appendicoliths were identified in 134/324 patients, of which 75 had complicated appendicitis. Among 190 patients without appendicoliths, 52 had complicated appendicitis. An appendicolith was independently associated with complicated appendicitis (adjusted odds ratio 2.289; 95% CI: 1.343–3.902; *p* = 0.002). The larger minimum diameter was significantly associated with complication. The 4.5-/6.0-mm cutoffs for minimum and maximum diameters of appendicoliths demonstrated 82.7%/85.3% sensitivity and 35.6%/33.9% specificity in predicting complications. The PVP alone had 82.1–88.1% sensitivity, respectively per patient and per appendicolith, and a 100% specificity in the detection of appendicoliths, as compared with combined noncontrast and PVP. PVP overlooked 28/237 appendicoliths (11.8%) corresponding to 24/134 patients (17.9%). Of the 24 patients with overlooked appendicoliths, 16 had complicated appendicitis but 14 were correctly categorized by findings other than appendicoliths. In total, 2/127 patients (1.6%) with complicated appendicitis were misdiagnosed as having uncomplicated appendicitis.

**Conclusions:**

Appendicoliths in acute appendicitis were strongly associated with complications. While PVP overlooked some appendicoliths, only 1.6% of complicated appendicitis were misclassified when considering other CT findings.

**Clinical relevance statement:**

This study found a strong association between appendicoliths and complications. Its presence may preclude conservative management. Although portovenous-phase CT overlooked some appendicoliths, the combination with other CT findings allowed correct classification in a vast majority of cases.

**Key Points:**

• *Accurate identification of appendicoliths is crucial for nonoperative management decisions in adult acute appendicitis.*

• *Appendicoliths are strongly associated with complications in adult acute appendicitis.*

• *Portovenous-phase CT overlooked some appendicoliths, but only a small percentage of patients with complicated appendicitis were misclassified when considering other CT findings.*

**Supplementary Information:**

The online version contains supplementary material available at 10.1007/s00330-023-10273-3.

## Introduction

Acute appendicitis is a common surgical emergency in adults, with a worldwide incidence estimated to be between 100 and 206 cases per 100,000 person-years [1]. Urgent appendectomy has been the traditional treatment approach for decades, with over 95% of cases managed surgically [2, 3]. However, nonoperative management (NOM) with antibiotic therapy has recently emerged as an alternative treatment strategy for uncomplicated appendicitis (i.e., those without gangrene or perforation), offering several benefits [4]. NOM failure and recurrent appendicitis are reported in 12–39% of patients [5–8], which is a concern. Therefore, careful patient selection and monitoring is crucial when considering NOM as a treatment option for adult appendicitis.

An appendicolith, a calcific material within the appendix seen on imaging studies [9, 10], has consistently been associated with complicated appendicitis in clinical, imaging, and pathological studies [5, 11–14]. Appendicoliths have been identified as independent predictors of failed NOM and recurrent appendicitis in patients with uncomplicated disease [15–18]. As a result, the presence of appendicolith in adult appendicitis may exclude patients from NOM [19, 20]. Recently, the World Society of Emergency Surgery [21] issued a guideline cautioning against nonsurgical treatment of appendicolith appendicitis. However, not all cases of appendicolith appendicitis are complicated, with appendicoliths present in 13.8–23.0% of those with acute uncomplicated appendicitis [17, 22]. Other features of appendicoliths, such as diameter and location, have been suggested in the study of Ishiyama et al as predictors of complication [23]. However, it remains uncertain if appendicolith characteristics could be used as selection criteria allowing NOM in a subset of patients with apparently uncomplicated appendicitis. Therefore, our study aimed to investigate the association between appendicoliths and complicated appendicitis and to identify differences in appendicolith characteristics between patients with complicated and uncomplicated appendicitis. Another aim was to explore the accuracy of portovenous-phase CT alone in the detection of appendicoliths compared to the combination of noncontrast and portovenous phases.

## Materials and methods

### Study design and patient selection

**Fig. 1:**
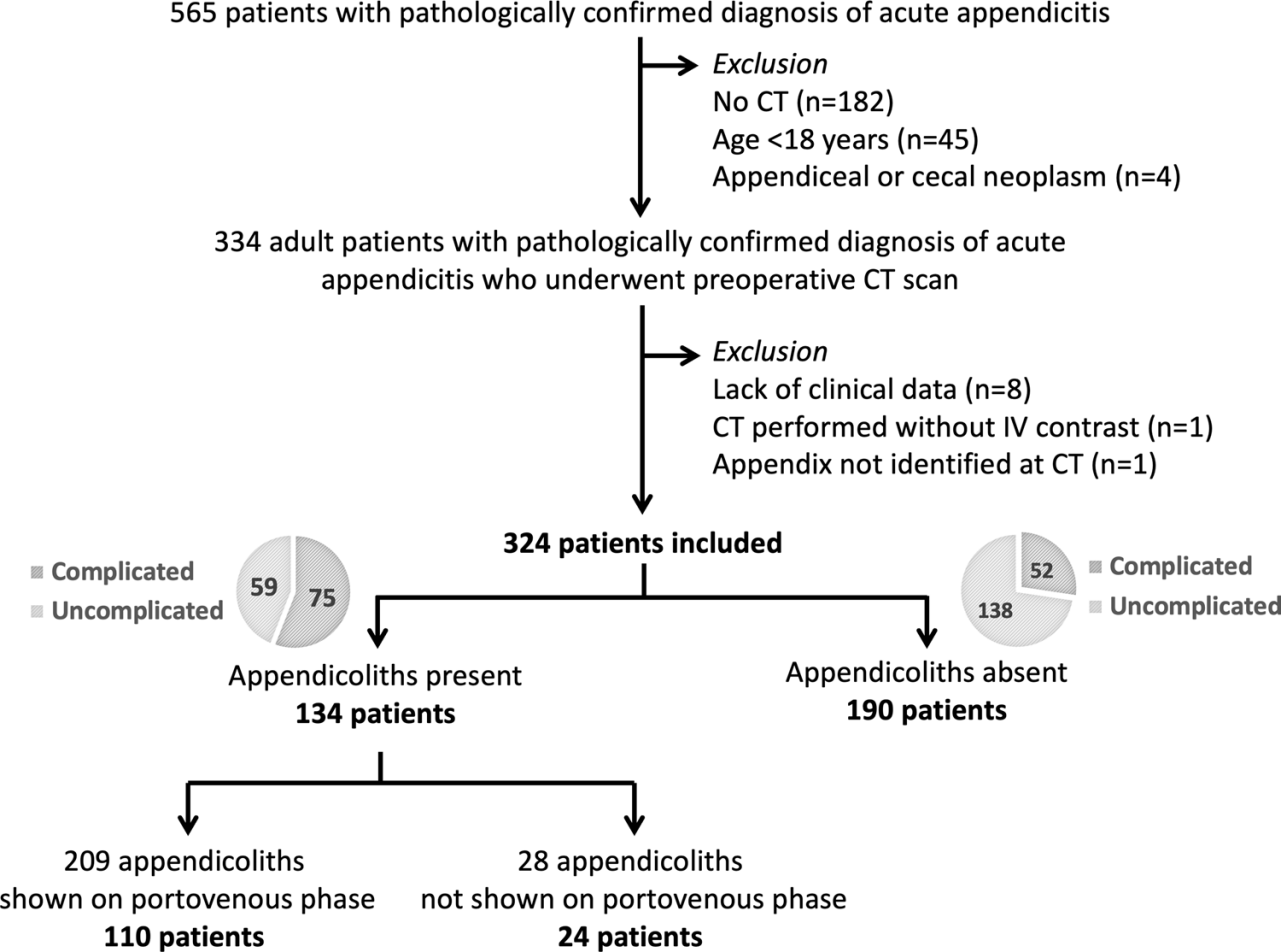
Flowchart of patient inclusion

This retrospective cross-sectional investigation was performed at a tertiary-care urban academic hospital, which has a capacity of 2200 beds. The hospital’s Institutional Review Board approved the study (protocol no. SIRB 198/2564 (IRB1)) and waived the requirement for informed consent due to its retrospective nature. Consecutive adult patients who underwent appendectomy with pathologically confirmed diagnosis of acute appendicitis and available preoperative CT were included. Patients were excluded if they had no clinical data available (*n* = 8), had CT performed without intravenous contrast (*n* = 1), or if the appendix was not identified on CT (*n* = 1). Note that while our previous investigation of different objective and endpoint [24] included a subset of 201 patients from this cohort, this investigation analyzed all 324 patients with available preoperative CT, which met the sample size calculated initially based on prevalence of appendicoliths of at least 30% with 95% confidence level and 5% allowable error. The flowchart of patient inclusion is provided in Fig. 1.

### Clinical data, image acquisition, reinterpretation, and definitions

**Fig. 2:**
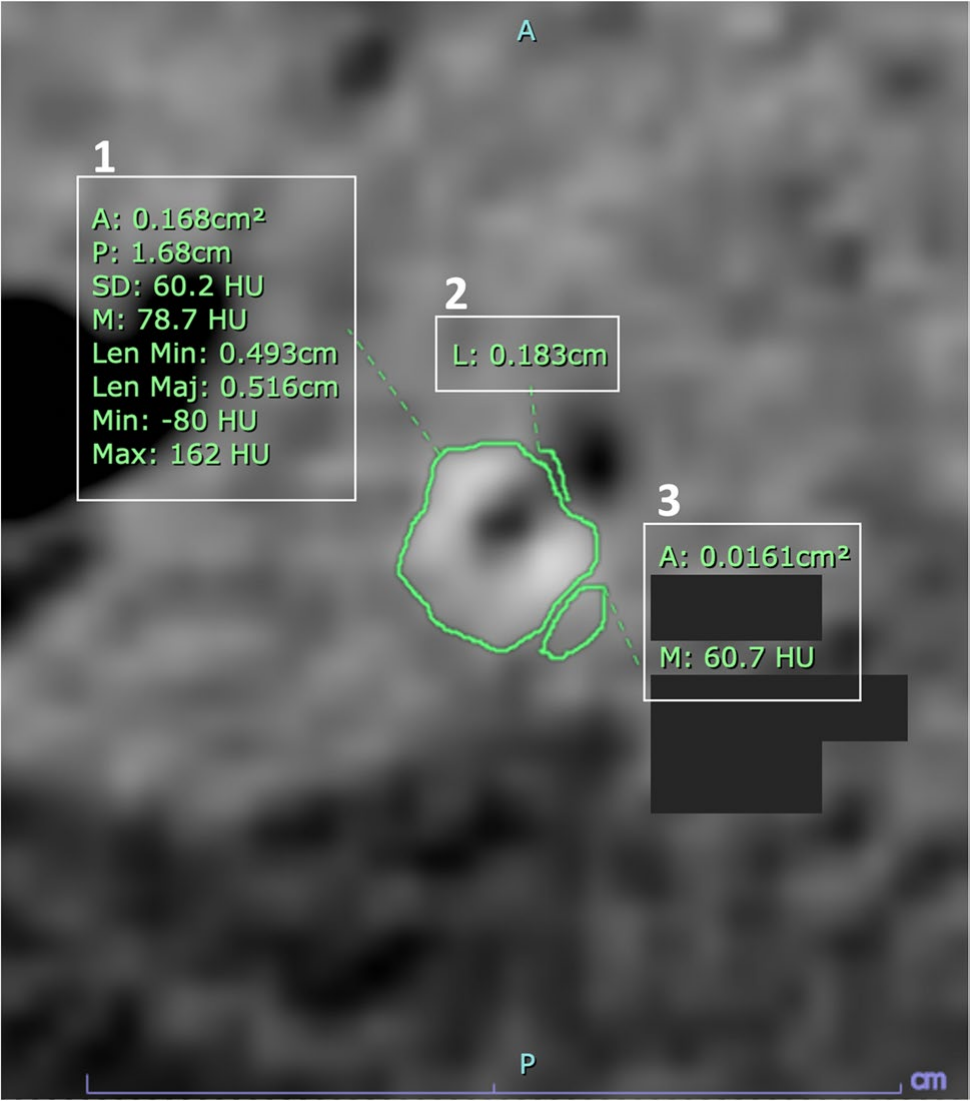
Measurements of appendicoliths (1), perimeter of air abutting appendicoliths (2), and soft tissue to the side of appendicoliths (3) using PACS tool. A, area (cm.^2^); L, length (cm); Len Max, maximum length (cm); Len Min, minimum length (cm); M, mean Hounsfield unit (HU); Max, maximum HU; Min, minimum HU; P, perimeter (cm); SD, standard deviation (HU)

Demographic data, time points (among symptoms, CT, and treatments), hospital length of stay, signs and symptoms, laboratory data, Alvarado score, type of appendectomy, and operative and pathological results were collected from the electronic medical records. CT scans were performed on one of our three multidetector scanners (64-slice LightSpeed VCT, 64-slice Discovery CT750 HD, or 256-slice Revolution CT, all from GE Healthcare). The scan coverage included from either the top of hemidiaphragms or kidneys to the pubic symphysis. They were performed without intravenous contrast (“noncontrast” phase), followed by administration of nonionic contrast medium at a rate of 2 mL/s, volume of 100 mL or 2 mL/kg via injectors, and a scan delay of 70–80 s (“portovenous” phase). The scan parameters were as follows: 120 kVp and 300 mAs for 64-MDCT or 250 mAs for 256-MDCT, respectively. Oral and rectal contrast media were not administered. Images of 1.25-mm slice thickness in both the noncontrast and portovenous phases were sent to Picture Archiving and Communication Systems for viewing. Two radiologists (one emergency and another abdominal subspecialists, both with 20 years of experience) independently reviewed the portovenous phase first for presence of appendicoliths. If an appendicolith was absent in this phase, the noncontrast phase was then evaluated. When an appendicolith was present, the number, signs of obstruction, and location were assessed. All discrepancies were resolved by a consensus. The detailed CT appearances of appendicitis in patients with appendicoliths shown only on the noncontrast phase but not the portovenous phase were re-reviewed using the same method. The measurements of appendicoliths and its surrounding tissues for size and CT numbers were performed on noncontrast-phase images by an emergency radiologist with a 20-year experience (example in Fig. 2). The measurements of appendix diameter were performed on axial portovenous phase CT images. The definitions of CT findings and measurements are provided in Supplementary Material 1.

### Categorization of appendicoliths by machine learning

Axial CT images in a Digital Imaging and Communications in Medicine format were selected at the mid-point of each appendicolith and set to a specific window level and window width of 60 and 225 Hounsfield units, respectively. A Portable Network Graphics image of each appendicolith was obtained and then manually segmented. Because the largest appendicolith did not exceed 36 pixels, the 36 × 36 pixel cropped images centered on the appendicolith were obtained. Any smaller images were resized to 36 × 36 pixels.

We adopted the semantic clustering by adopting nearest neighbors model [25] because, at the time of writing, it was one of the state-of-the-art models on unsupervised image classification and image clustering (https://paperswithcode.com). This model consists of the following steps:

1. Representation learning for semantic clustering, which was similar to contrastive learning.
2. A semantic clustering loss. This technique trained a classifier model with the goal of assigning instances neighboring each other to the same class while maximizing entropy, which kept other clusters away in the representation space. Since the number of appropriate clusters was needed to be determined beforehand, we used the elbow method and silhouette method [26] with K-means clustering on the representation space obtained in step 1. We also applied principal component analysis [27]. This resulted in “2” as the most appropriate number of clusters in this investigation.
3. Fine-tuning through self-labeling.

To improve the performance of medical image classification, especially on a small dataset, we also utilized transfer learning [28].

**Fig. 3:**
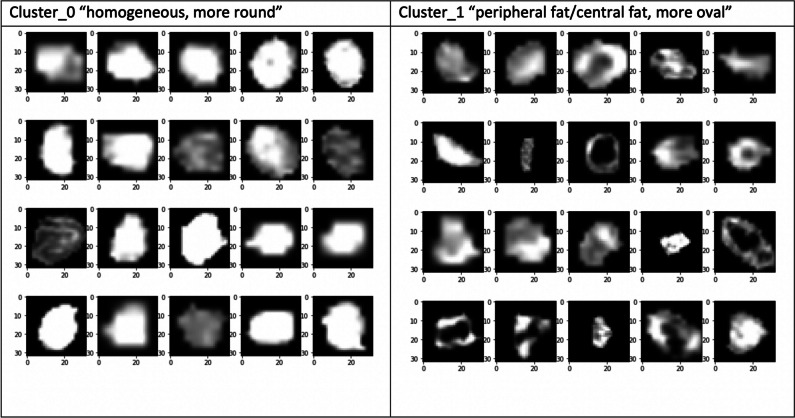
Type of appendicoliths suggested by machine learning algorithm

By applying this methodology, two categories or types of appendicoliths were identified, as presented in Fig. 3, which we referred to as type 0 (homogeneous and rounded appendicoliths) and type 1 (heterogeneous appendicoliths with central or peripheral hypoattenuation, and oval). Subsequently, “representative” appendicoliths of all patients were independently classified by a 2nd-year radiology resident and a 1st-year radiology resident based on this categorization, and any discrepancies were resolved by an emergency radiologist with a 20-year experience. The results (“type of appendicolith”) were then used as a part of CT characteristics of appendicoliths.

### Reference standards

In this study, noncontrast-phase CT was regarded as the diagnostic reference for appendicoliths, which were defined as hyperattenuating foci with a diameter > 2 mm located either inside the appendiceal lumen or outside in fluid or fluid collection [10, 12]. The diagnosis of acute appendicitis was based on histopathological results, while complicated appendicitis, including gangrene and perforation, was diagnosed by either histopathology or surgical operative findings [29]. Histopathology was used to diagnose gangrene, while either histopathology or surgical operative findings were employed to diagnose perforation.

### Statistical analysis

Descriptive statistics were used to analyze both qualitative and quantitative data. Categorical variables were presented as numbers or percentages, while continuous data were reported as either mean (standard deviation) or median (range) depending on their normal or skewed distribution.

Inferential statistics were employed to compare the differences between the two groups (patients without vs. with appendicolith, complicated vs. uncomplicated appendicitis, and appendicoliths not shown vs. shown on portovenous phase). The Pearson chi-square test, Yates continuity correction, or Fisher exact test was utilized for categorical variables, and the independent-sample *t*-test or Mann–Whitney *U* test was used for continuous variables with means or medians, respectively. Logistic regression approach or marginal logistic regression using the *generalized estimating equations* (GEE) model in order to account for correlation between appendicoliths in the same patient was applied for univariable and multivariable analyses to determine the independent predictors of the presence of appendicolith or non-detection of appendicoliths in the portovenous phase computed tomography. The odds ratio (OR) and adjusted OR (aOR) *with corresponding* 95% confidence intervals (95% CI) were used to identify the strength and direction of their association. The selection of factors into the multivariable model was based on a *p* value of less than 0.1 in a univariable model. Cutoff values of potential continuous variables that may predict complicated appendicitis in patients with appendicolith were derived. The area under the receiver operating characteristics (ROC) curve (AUC) with the corresponding 95% confidence interval (95% CI) was considered as a summary measure for discrimination.

The diagnostic performance of portovenous-phase CT in diagnosing appendicoliths was evaluated using sensitivity, specificity, positive likelihood ratio, negative likelihood ratio, positive predictive value, negative predictive value, and accuracy. All analyses were performed using the IBM SPSS Statistics for Windows Version 26.0 and considering *a statistical significance of a p value less than 0.05*.

## Results

The study included 324 patients with a median age of 54 years (range, 18–94) and a female predominance (65.4%). The median Alvarado score was 7 (range, 1–10), and the mean appendix diameter measured on CT was 12 mm (SD, 2.9). Of the 324 patients, 127 (39.2%) had complicated appendicitis. Table 1 provides information and comparison between patients with and without appendicoliths. Univariable and multivariable analyses (Table 2) identified 5 independent factors associated with appendicoliths, including a shorter duration from emergency room arrival to antibiotics (aOR = 0.926; 95% CI: 0.865–0.991), rebound tenderness (aOR = 2.067; 95% CI: 1.258–3.398), larger appendix diameter on CT (aOR = 1.140; 95% CI: 1.039–1.250), longer length of stay for initial admission (aOR = 1.124; 95% CI: 1.007–1.253), and presence of complicated appendicitis (aOR = 2.289; 95% CI: 1.343–3.902).

**Table 1:** Patient characteristics between those with and without appendicoliths (*n* = 324)

Factors	All patients (*n* = 324)	With appendicolith (*n* = 134)	Without appendicolith (*n* = 190)	*p* values
Demographics				
Age (years; mean, SD)	51.9 (19.6)	53.7 (18.4)	50.7 (20.3)	0.178
Age intervals (*n*, %)				
18–39 years	104 (32.1)	36 (26.9)	68 (35.8)	0.135
40–59 years	88 (27.2)	43 (32.1)	45 (23.7)	
60 years and older	132 (40.7)	55 (41.0)	77 (40.5)	
Female (*n*, %)	212 (65.4)	87 (64.9)	125 (65.8)	0.966
BMI (kg/m^2^; median, range)	23.6 (12.7, 48. 9)	23.4 (12.7, 36.1)	23.8 (14.5, 48. 9)	0.865
Duration from onset to ER arrival (*n* = 322) ≥ 24 h	197 (61.2)	92 (68.7)	105 (55.9)	*0.027*
Durations (hrs; median, range)				
Onset to ER arrival (*n* = 322)	24 (2, 480)	24 (3, 240)	24 (2, 480)	*0.002*
ER arrival to CT (*n* = 320)	5.2 (0.2, 82.1)	5.1 (0.2, 42.4)	5.3 (0.5, 82.1)	0.247
CT to surgery (*n* = 314)	4.0 (0.3, 74.1)	4.0 (0.3, 47.1)	4.1 (1.1, 74.1)	0.721
ER arrival to surgery (*n* = 316)	9.6 (3.0, 87.6)	9.3 (3.0, 52.5)	10.1 (3.2, 87.6)	0.412
ER arrival to antibiotics (*n* = 321)	6.5 (0, 29.4)	6.4 (0, 20.4)	7.1 (0.4, 29.4)	*0.012*
Length of stay (days)	3 (1, 44)	4 (1, 44)	2 (1, 36)	< *0.001*
Signs and symptoms				
RLQ pain (*n*, %)	313 (96.6)	131 (97.8)	182 (95.8)	0.535
Temp (°C; mean, SD)	37.3 (0.8)	37.3 (0.8)	37.3 (0.8)	0.915
Rebound tenderness (*n*, %)	161 (49.7)	80 (59.7)	81 (42.6)	*0.004*
Migratory pain (*n*, %)	143 (44.1)	51 (38.1)	92 (48.4)	0.083
Anorexia (*n*, %)	158 (48.8)	68 (50.7)	90 (47.4)	0.627
Nausea and vomiting (*n*, %)	189 (58.3)	78 (58.2)	111 (58.4)	0.970
Labs (median, range)				
White blood cell counts (× 10^9^ cells/L)	13.2 (0.7, 29.2)	12.7 (3.0, 29.2)	13.3 (0.7, 24.7)	0.633
Neutrophils (%) (*n* = 322)	82.6 (4.0, 97.0)	84.1 (21.0, 96.0)	81.4 (4.0, 97.0)	*0.006*
Absolute neutrophils (× 10^9^ cells/L) (*n* = 322)	10.6 (0.03, 26.6)	10.5 (1.5, 26.6)	10.7 (0.03, 22.3)	0.766
Eosinophils (%)	0.2 (0, 15.1)	0.1 (0, 9.1)	0.3 (0, 15.1)	*0.001*
Absolute eosinophils (× 10^9^ cells/L)	0.03 (0, 1.4)	0.02 (0, 1.3)	0.04 (0, 1.4)	*0.002*
Alvarado score (median, range)	7 (1, 10)	7 (3, 10)	7 (1, 10)	0.508
Appendix diameter on CT (mm; mean, SD)	12.0 (2.9)	12.8 (3.0)	11.4 (2.7)	< *0.001*
Type of appendectomy (*n*, %)				0.083
Open	273 (84.2)	120 (89.6)	153 (80.5)	
Laparoscopic	42 (13.0)	11 (8.2)	31 (16.3)	
Delayed	9 (2.8)	3 (2.2)	6 (3.2)	
Presence of complicated appendicitis (*n*, %)	127 (39.2)	75 (56.0)	52 (27.4)	< *0.001*

*p* values of < 0.05 are marked with italics

**Table 2:** Multivariable analysis of factors associated with presence of appendicoliths (*n* = 324)

Factors	Univariable model		Multivariable model	
	Unadjusted OR (95% CI)	*p* values	Adjusted OR (95% CI)	*p* values
ER arrival to antibiotics	0.910 (0.855, 0.968)	0.003	0.926 (0.865, 0.991)	*0.026*
Rebound tenderness	1.994 (1.272, 3.124)	0.003	2.067 (1.258, 3.398)	*0.004*
Appendix diameter on CT	1.196 (1.099, 1.302)	< 0.001	1.140 (1.039, 1.250)	*0.006*
Length of stay for initial admission	1.140 (1.045, 1.244)	0.003	1.124 (1.007, 1.253)	*0.036*
Presence of complicated appendicitis	3.374 (2.115, 5.381)	< 0.001	2.289 (1.343, 3.902)	*0.002*

The independent variables with *p* value < 0.10 in simple logistic regression model and without multicollinearity were included in multivariable analysis

*p* values of < 0.05 are marked with italics

*OR*, odds ratio

A total of 134 patients had at least one appendicolith, with 75 patients having complicated appendicitis and 59 having uncomplicated appendicitis. Except for the minimum diameter of appendicoliths, other CT characteristics (including type, number, presence of obstruction, location, size, and CT attenuation; Table 3) showed no significant differences between the appendicoliths found in patients with complicated vs. uncomplicated appendicitis. The areas under the ROC curve of the minimum and maximum diameters of appendicolith were 0.607 (95% CI; 0.510–0.704) and 0.566 (95% CI; 0.466–0.666), with *p* values of 0.03 and 0.19, respectively. The optimal cutoff value of the minimum diameter was identified at 4.5 mm (*p* = 0.03), which yielded 62 true positives, 38 false positives, 13 false negatives, and 21 true negatives. The sensitivity, specificity, positive predictive value, and negative predictive value with their respective 95% CI were 82.7% (72.6–89.6%), 35.6% (24.6–48.3%), 62.0% (52.2–70.9%), and 61.8% (45.0–76.1%), respectively. The optimal cutoff value of the maximum diameter was identified at 6.0 mm (*p* = 0.02), which yielded 64 true positives, 39 false positives, 11 false negatives, and 20 true negatives. The sensitivity, specificity, positive predictive value, and negative predictive value with their respective 95% CI were 85.3% (75.6–91.6%), 33.9% (23.1–46.6%), 62.1% (52.5–70.9%), and 64.5% (46.9–78.9%), respectively.

**Table 3:** Computed tomographic characteristics of appendicoliths in patients with complicated and uncomplicated appendicitis (*n* = 134)*

Characteristics	All patients (*n* = 134)	Complicated appendicitis (*n* = 75)	Uncomplicated appendicitis (*n* = 59)	*p* values
Type of appendicolith				0.766
Type 0	53 (39.6)	31 (41.3)	22 (37.3)	
Type 1	81 (60.4)	44 (58.7)	37 (62.7)	
Number of appendicolith per patient				0.631
1	82 (61.2)	46 (61.3)	36 (61.0)	
2	24 (17.9)	13 (17.3)	11 (18.6)	
3	16 (11.9)	10 (13.3)	6 (10.2)	
4	5 (3.7)	1 (1.3)	4 (6.8)	
5	4 (3.0)	3 (4.0)	1 (1.7)	
6	2 (1.5)	1 (1.3)	1 (1.7)	
8	1 (0.7)	1 (1.3)	0 (0)	
Obstructive appendicolith (*n*, %)	87 (64.9)	50 (66.7)	37 (62.7)	0.769
Location				0.253
Proximal	85 (63.9)	49 (65.3)	36 (62.1)	
Mid	29 (21.8)	13 (17.3)	16 (27.6)	
Distal	19 (14.3)	13 (17.3)	6 (10.3)	
Size				
Maximum diameter (mm; median, range)	8.2 (2.4, 24.1)	8.7 (2.4, 24.1)	8.0 (2.7, 16.4)	0.191
Maximum diameter ≥ 6.0 mm	103 (76.9)	64 (85.3)	39 (66.1)	*0.016*
Minimum diameter (mm; median, range)	6.0 (1.7, 12.1)	6.4 (1.7, 11.5)	5.7 (1.9, 12.1)	*0.034*
Minimum diameter ≥ 4.5 mm	100 (74.6)	62 (82.7)	38 (64.4)	*0.027*
Maximum cross-sectional area (mm^2^; median, range)	4.1 (1.0, 80.3)	4.4 (1, 80.3)	3.8 (1.0, 70.5)	0.352
Ratio between appendicolith maximum diameter and appendix diameter (median, range)	0.7 (0.2, 2.7)	0.7 (0.2, 2.7)	0.6 (0.2, 1.5)	0.352
Ratio between appendicolith minimum diameter and appendix diameter (mm; median, range)	0.5 (0.1, 2.6)	0.5 (0.1, 2.6)	0.5 (0.1, 0.8)	0.059
Perimeter (mm; median, range)	25.2 (7.3, 62.7)	26.4 (7.3, 62.7)	23.8 (7.5, 47.8)	0.081
CT attenuation (HU)				
Mean (median, range)	119.3 (− 3.6, 1528.0)	118 (− 4, 1528)	123.9 (7.5, 1195.0)	0.603
Standard deviation (median, range)	55.3 (1.1, 3183.0)	58.7 (1.1, 900.2)	49.8 (17.1, 3183.0)	0.400
Maximum HU (median, range)	224.5 (− 155, 3071)	225 (113, 3071)	224 (− 155, 3071)	0.946
Minimum HU (median, range)	19.5 (− 370, 162)	17 (− 370, 147)	23 (− 210, 162)	0.093
Difference between min and max HU (median, range)	224.5 (− 91, 3219)	250 (85, 3165)	204 (− 91, 3219)	0.120
CT attenuation of surrounding soft tissues (HU)				
Noncontrast phase (median, range)	80.7 (− 44.6, 1498.4)	79.6 (− 44.6, 1498.4)	81.6 (− 35.2, 1134.0)	0.628
Portovenous phase (median, range)	69.2 (− 98.7, 1479.6)	61.1 (− 98.7, 1479.6)	70.6 (− 35.1, 1182.1)	0.659

^*^In patients with multiple appendicoliths, only the representative appendicolith was used for the analysis

*p *values of < 0.05 are marked with italics

*HU*, Hounsfield unit; *NC*, noncontrast; *PVP*, portovenous phase

A total of 237 appendicoliths were found in these 134 patients. Sensitivities in the detection of appendicolith on the portovenous-phase CT were 88.2% per appendicolith and 82.1% per patient. There were 28 false negatives (per appendicolith) and 24 (per patient) (Table 4).

**Table 4:** Diagnostic performance of portovenous phase computed tomography in the detection of appendicoliths using combined noncontrast and portovenous phases as a reference standard

	Per appendicolith (*n* = 427)	Per patient (*n* = 324)
True positive	209	110
False positive	0	0
False negative	28	24
True negative	190	190
Sensitivity (%)	88.2 (83.4, 92.0)	82.1 (74.7, 87.7)
Specificity (%)	100 (98.1, 100)	100 (98.0, 100)
Positive likelihood ratio	N/A (44.5, ∞)	N/A (41.4, ∞)
Negative likelihood ratio	0.12 (0.08, 0.17)	0.18 (0.12, 0.26)
Disease prevalence (%)	55.5 (50.7, 60.3)	41.4 (36.1, 46.8)
Positive predictive value (%)	100 (98.2, 100)	100 (96.6, 100)
Negative predictive value (%)	87.2 (82.7, 90.6)	88.8 (83.9, 92.3)
Accuracy (%)	93.4 (90.7, 95.6)	92.6 (89.2, 95.0)

Values in brackets represent 95% confidence interval

Univariable and multivariable analyses (Supplementary Material 2 and Table 5) revealed four factors associated with false-negative results on the portovenous-phase CT. These included appendicoliths with a homogenous appearance (aOR = 6.803; 95% CI: 1.202–38.462), smaller minimum diameter (aOR = 0.034; 95% CI: 0.002–0.591), smaller differences between maximum and minimum CT attenuation (aOR = 0.994; 95% CI: 0.990–0.999), and smaller differences between CT attenuation of appendicolith and surrounding soft tissues (aOR = 0.966; 95% CI: 0.943–0.989).

**Table 5:** Multivariable analysis of factors associated with non-detection of appendicolith in the portovenous phase computed tomography of adult patients with acute appendicitis (*n* = 237)

Factors	Univariable model		Multivariable model	
	Unadjusted OR (95% CI)	*p* value	Adjusted OR (95% CI)	*p* value
Complicated appendicitis	3.347 (1.387, 8.075)	0.007	1.109 (0.298, 4.131)	0.877
Homogenous appearance of appendicoliths	2.976 (0.822, 10.778)	0.097	6.803 (1.202, 38.462)	*0.030*
Mean HU of appendicoliths	0.981 (0.973, 0.989)	< 0.001	0.994 (0.963, 1.027)	0.730
Minimum diameter of appendicoliths	0.179 (0.029, 1.103)	0.064	0.034 (0.002, 0.591)	*0.020*
Difference between maximum and minimum HU	0.990 (0.980, 1.000)	0.043	0.994 (0.990, 0.999)	*0.014*
Difference between HU of appendicoliths and surrounding soft tissues in NC	0.981 (0.971, 0.990)	< 0.001	0.987 (0.963, 1.012)	0.317
Difference between HU of appendicoliths and surrounding soft tissues in PVP	0.981 (0.970, 0.992)	0.001	0.966 (0.943, 0.989)	*0.004*

The independent variables with *p* value < 0.10 in simple marginal logistic regression using a GEE model and without multicollinearity were included in the multivariable analysis

*p* values of < 0.05 are marked with italics

*HU*, Hounsfield unit; *NC*, noncontrast; *PVP*, portovenous phase

**Fig. 4:**
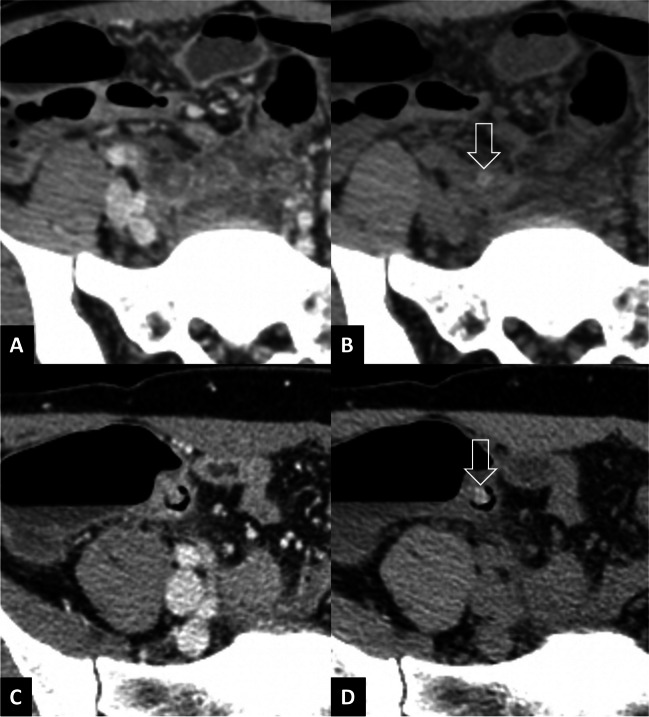
Example of two cases with appendicoliths (arrows) undetected on portovenous-phase CT (**A**, **C**) but visible on noncontrast CT (**B**, **D**)

Twenty-four patients had 28 appendicoliths not detected on the portovenous phase (Fig. 4, Supplementary Material 3). Among these 24 patients, 16 had complicated appendicitis as confirmed by histopathology or surgical operative findings. CT correctly identified complications in 14 patients, while 5 were correctly identified as not having complications. There were 2 false negatives and 3 false positives during the re-review of CT images.

## Discussion

This investigation highlights the importance of identifying appendicoliths in adult appendicitis due to their association with complications, like gangrene or perforation, as well as significantly longer hospital stays. The only CT characteristic of appendicolith significantly associated with increased likelihood of complicated appendicitis was the larger minimum diameter of appendicoliths. The sensitivities of portovenous-phase CT in detecting appendicoliths were 82.1% per patient and 88.2% per appendicolith. Unsurprisingly, these undetected appendicoliths had a small minimum diameter and a homogeneous appearance, and were less distinguishable from surrounding soft tissues. These overlooked appendicoliths, however, resulted in misclassification of acute appendicitis in only 1.6% of patients because other CT findings of complication were present in the portovenous-phase CT.

### Association of appendicoliths with complicated appendicitis

The presence of appendicoliths has consistently been associated with complicated appendicitis in several studies. These studies suggest that appendicolith appendicitis has a higher severity of inflammation in pathological specimens than that without appendicoliths [14], a higher severity of inflammation on imaging [10], a higher prevalence of perforation [30], and an increased rate of failed NOM and complications [5, 11, 12, 18, 22]. Our investigation supports this notion by revealing that patients with appendicolith appendicitis were approximately twice as likely to have complicated appendicitis at pathology. Furthermore, we associated appendicoliths with markers of increased severity, such as rebound tenderness, a larger appendix diameter on CT, and a longer length of hospital stay, which are in line with previous studies [12, 22].

Although appendicoliths were found to be strongly associated with complicated appendicitis, several investigations reported a prevalence of appendicoliths in pathologically proven uncomplicated appendicitis of 13.8 to 23.0% [17, 22]. Our investigation showed a higher prevalence of 44% (59 out of 134 patients). Previous studies have suggested that appendicolith size [12, 23, 31] and location [23, 32] are associated with complications, and our investigation found that only the minimum diameter of appendicoliths was independently associated with complicated appendicitis. It is unclear why the minimum diameter, instead of the maximum diameter, is a predictor of complicated appendicitis. However, cutoff values (4.5 mm for the minimum diameter and 6.0 mm for the maximum diameter of appendicoliths) could be established for both diameters with reasonable AUCs and relatively high sensitivities but poor specificities. Previous investigations [12, 23, 31] identified a maximum diameter as an independent predictor of complicated appendicitis. A 5-mm cutoff value was reported as useful for suggesting nonoperative management [12] or prediction of complicated appendicitis [23, 31], while a 10-mm cutoff value was suggested as a cutoff for appendectomy [12]. As these investigations did not typically collect the minimum diameter, it is difficult to determine whether it would be a more accurate representation of the appendicolith’s diameter.

### Diagnostic performance of portovenous-phase CT in the detection of appendicolith

The accurate noninvasive detection of appendicoliths in adults with appendicitis becomes important for determining whether NOM is a feasible option in an otherwise uncomplicated case. The diagnostic performance of contrast-enhanced CT in the detection of appendicoliths had been explored in detail in a few investigations that used surgical specimens and histopathology as a reference standard [30, 33]. These investigations identified a wide range of sensitivity (21–81%; overall 56%), specificity (78–96%; overall 86%), and radiologists’ agreement (kappa; 0.48–0.83) [33]. However, we believe that the poor CT performance in the detection of appendicolith in these reports stemmed from the radiologic definition of appendicoliths as a “calcific” deposit [9, 10], which categorically excludes noncalcific intraluminal contents within an appendix from being classified as appendicolith. This leads to a lower sensitivity of CT in this regard. In addition, using histopathology as a reference standard has limitations, including difficulties in differentiating between a fecalith and a calcific counterpart (i.e., appendicolith as per CT definition), variances in evaluating appendiceal specimen vs in situ CT, and loss of appendiceal contents during transfer of the specimen [14].

To address these limitations and facilitate practical prospective patient management, we utilized a combined noncontrast and portovenous-phase CT as a reference for identifying appendicoliths in our study, recognizing its imperfections. We used a prespecified standard definition that had thresholds for both size (> 2 mm; to allow accurate and reproducible detection) and CT attenuation (visibly higher than surrounding tissue) [9, 10] to reduce bias. Using CT as a reference also reflects real-world application as this is the case for many trials [5–8]. However, this approach comes at a cost of not knowing the implication of “noncalcified” contents within the inflamed appendix.

Previous studies have reported a prevalence of appendicoliths in adult appendicitis ranging from 33 to 38.7% [10, 34, 35]. However, our study found a higher prevalence at 41.4%. This difference may be attributed to our use of a broader definition of appendicoliths as high-attenuation materials rather than strict calcifications, and the utilization of combined noncontrast and portovenous-phase CT instead of a single portovenous-phase CT, which is typically used in previous investigations. Noncontrast CT is known to be superior to portovenous-phase CT in detecting calcifications, as demonstrated in studies on urolithiasis, cholelithiasis, and choledocholithiasis [36, 37]. Calcifications stand out more obviously relative to surrounding soft tissues in the noncontrast phase than in the portovenous phase. Our multivariable analysis of factors associated with appendicoliths not detected on portovenous-phase CT revealed that their CT attenuation was significantly closer to that of surrounding soft tissues. Additionally, they had a more homogeneous appearance (both visually and by measurement of differences between maximum and minimum CT attenuation), and a smaller minimum diameter.

Using this combined noncontrast and portovenous-phase CT, we identified a higher prevalence of appendicoliths, with 11.8% more appendicoliths per appendicolith and 17.9% more appendicoliths per patient compared to using portovenous-phase CT alone. Strict adherence to the World Society of Emergency Surgery guideline would result in a higher number of appendicitis cases being excluded from NOM due to the presence of appendicoliths. It is important to acknowledge that implementing this guideline may lead to some patients with uncomplicated appendicitis being ineligible for NOM. This is to ensure a safe practice of recommending appendectomy for patients with uncomplicated appendicitis (with appendicolith) rather than resorting to NOM for those with complicated appendicitis. In fact, even when the portovenous-phase CT alone was used to select patient for NOM, the overwhelming majority of patients with proven complicated appendicitis would still be correctly identified due to the presence of CT findings other than appendicoliths. In our cohort, only two out of 127 patients (1.6%) with complicated appendicitis would be misdiagnosed as having uncomplicated appendicitis on CT. Our investigation still supports the use of portovenous-phase CT, even when used alone without noncontrast phase, as it can still identify almost all cases of complicated appendicitis through findings other than appendicoliths such as fluid collections and extraluminal air.

The study has several limitations. Firstly, it was a retrospective single-center study with a relatively small sample size (although it did reach a precalculated level). Additionally, a large proportion of our patients consisted of the elder population, which may explain the high rate of complication (almost 40%) detected in this investigation [38]. Secondly, many patients with appendicitis were excluded due to the use of alternative diagnostic methods, such as preoperative ultrasound or outside-hospital CT, or because they directly underwent surgery. These may affect the proportion of patients with and without complication, and limit the generalizability of the findings to other populations or settings. Since appendectomy remains the standard of care for appendicitis in our clinical practice, we believe that the likelihood of excluding uncomplicated appendicitis being treated with NOM is minimal. Thirdly, not all potential clinical confounders were collected, which limits our ability to confidently conclude on the association between certain clinical parameters and the presence of appendicoliths. Fourthly, while the study suggested that the minimum diameter of appendicolith was independently associated with complicated appendicitis, further studies are needed to confirm this finding. Fifthly, since the primary aim of NOM was to ensure that patients with complicated appendicitis were not mistakenly selected for NOM, identifying detailed appendicolith characteristics for their potential selection for NOM may be counterproductive. Lastly, while the study found that noncontrast-phase CT identifies more appendicoliths than the portovenous phase alone, the added value of identifying appendicolith may be limited by other CT findings that can direct patients to a complicated group (i.e., for appendectomy).

In conclusion, our study found a significant association between appendicoliths and complicated appendicitis—in particular those with a larger minimum diameter. While the sensitivity of portovenous-phase CT in detecting appendicoliths was modest compared to combined noncontrast and portovenous-phase CT, the portovenous phase alone was sufficient in accurately identifying complicated appendicitis through the presence of other CT findings. Consequently, the routine use of combined noncontrast and portovenous-phase scans cannot be recommended. However, in older patients with suspected acute appendicitis under consideration of NOM, the inclusion of a noncontrast phase may be justified. Detecting appendicoliths in this patient subset can provide an objective means to diagnose complicated appendicitis, which is associated with increased morbidity and mortality as age advances [38]. Furthermore, the lower risk of radiation-related cancer in older patients [39] likely outweighs the potential benefits of characterizing complicated appendicitis, making the inclusion of a noncontrast phase more justifiable for this age group. While our results provided insight into the selection of patients for NOM, further research is needed to validate the significance of appendicolith size in predicting complicated appendicitis. New techniques like dual-energy CT may offer direct findings of complicated appendicitis but the potential of virtual noncontrast images as a substitute for true noncontrast images, reducing radiation exposure, remains uncertain.

### Supplementary Information

Below is the link to the electronic supplementary material.

Supplementary file1 (PDF 227 KB)

## Abbreviations

aOR: Adjusted odds ratio
CI: Confidence intervals
CT: Computed tomography
GEE: Generalized estimating equations
HU: Hounsfield units
MDCT: Multidetector computed tomography
NOM: Nonoperative management
OR: Odds ratio

## Declarations

### Guarantor

The scientific guarantor of this publication is Rathachai Kaewlai.

### Study subjects or cohorts overlap

The current research on adult appendicitis and appendicoliths is an extension of a previous study conducted by our group and reported in 2021 (10.1186/s13244-021-01086-3), which included 201 patients. However, the current report provides new and expanded insights into the topic by including a total of 324 patients.

Our previous study had focused on differentiating between uncomplicated and complicated appendicitis, while the current report has a different focus. Specifically, the current report examines the characteristics of appendicoliths in adult appendicitis and their implications for diagnosis and treatment.

With an increased sample size of 324 patients, the current report is able to provide more detailed information on the topic. The findings of this research have important implications for clinical practice, as a better understanding of the characteristics of appendicoliths in adult appendicitis can lead to more accurate diagnoses and more effective treatments.

### Methodology

• Retrospective

• Cross-sectional study

• Performed at one institution
