# Supplementary material for: CT of appendicoliths in adult appendicitis: clinical significance and characteristics of overlooked cases
Source: Eur Radiol. 2023 Oct 14;34(4):2534–45. doi: 10.1007/s00330-023-10273-3 (PMC10957675; doi:10.1007/s00330-023-10273-3)
Supplement: Supplementary file 1 — Supplementary file1 (PDF 227 KB) [file 330_2023_10273_MOESM1_ESM.pdf]

CT of appendicoliths in adult appendicitis: clinical significance and characteristics of overlooked cases

Electronic Supplementary Material (ESM)

Eur Radiol (2023) Kaewlai R, Wongveerasin P, Lekanamongkol W et al.

Supplementary Material 1: Definitions of CT findings and measurements

**Findings or measurements**	**Definitions**
Appendix diameter	Outer-to-outer wall shortest diameter of appendix measured in the axial CT images that were perpendicular to the appendix's axis.
Appendicolith	A hyperattenuating focus with a diameter >2 mm located either inside the appendiceal lumen or outside in fluid or fluid collection.
Representative appendicolith	In a patient with >1 appendicoliths, the largest appendicolith in terms of maximum diameter was selected as representative of all appendicoliths in that patient.
Type of appendicolith	Please see the subsection "Categorization of appendicoliths by machine learning"
Obstructive appendicolith	Appendicolith with dilation of the upstream appendix diameter equal to or greater than that of the appendicolith itself
Location of appendicolith	The location was described as being in the proximal 1/3, middle 1/3, or distal 1/3 of the appendix based on its position relative to the length of the appendix.
Size of appendicolith	Determined by measuring appendicolith's maximum and minimum diameter, cross-sectional area, and perimeter.
CT attenuation of appendicolith	Determined by measuring appendicolith's median, maximum, and minimum values, and the difference between the maximum and minimum values.
CT attenuation of surrounding soft tissues	Determined by measuring surrounding soft tissues' median, maximum, and minimum values

Supplementary Material 2: Computed tomographic characteristics of appendicoliths not shown and shown on portovenous phase of adult patients with acute appendicitis (n=237)

**Characteristics**	**Appendicoliths not shown on portovenous phase (n=28)**	**Appendicoliths shown on portovenous phase (n=209)**	**p-values**
**Type of appendicitis**			0.750
Uncomplicated	11 (39.3)	93 (44.5)	
Complicated	17 (60.7)	116 (55.5)	
**Type of appendicolith**			0.263
Type 0 (homogeneous, rounded)	8 (28.6)	87 (41.6)	
Type 1 (heterogeneous, oval/oblong)	20 (71.4)	122 (58.4)	
**Obstructive appendicolith** (n, %)	14 (50.0)	149 (71.3)	*0.039*
**Homogeneous attenuation** (n, %)	25 (89.3)	154 (73.7)	0.117
**Location** (n, %)			
Proximal	13 (46.4)	95 (45.5)	0.481
Mid	10 (35.7)	57 (27.3)	
Distal	5 (17.9)	57 (27.3)	
**Size**			
Maximum diameter (cm; median, range)	0.6 (0.3, 1.4)	0.7 (0.2, 2.4)	0.114
Minimum diameter (cm; median, range)	0.4 (0.2, 0.8)	0.5 (0.2, 1.2)	0.098
Maximum cross-sectional area (cm^2; median, range) (n=233)	0.3 (0.1, 8.0)	0.4 (0.1, 9.9)	0.229
Ratio between appendicolith maximum diameter and appendix diameter (median, range)	0.05 (0.03, 0.27)	0.05 (0.02, 0.21)	0.622
Ratio between appendicolith minimum diameter and appendix diameter (median, range)	0.04 (0.01, 0.26)	0.04 (0.01, 0.09)	0.433
Perimeter (cm; median, range)	1.8 (0.8, 3.4)	2.1 (0.6, 6.3)	0.145
**CT attenuation (HU)**			
Mean (median, range)	87.9 (-3.6, 139.4)	128.1 (1.7, 1528.0)	*<0.001*
Standard deviation (median, range)	29.1 (17.1, 110.0)	53.2 (1.1, 3183.0)	*<0.001*
Maximum HU (median, range)	144 (113, 249)	238.5 (-155, 3071)	*<0.001*
Minimum HU (median, range)	23.5 (-362, 78)	24.0 (-370, 479)	0.606
Difference between maximum and minimum HU (median, range)	128.5 (72, 570)	223.5 (-91, 3219)	*<0.001*

**Perimeter of air around appendicolith** (median, range)			
Length (mm)	0 (0, 0.8)	0 (0, 1.6)	0.514
% of air compared with appendicolith	0 (0, 37.8)	0 (0, 77.1)	0.550
**HU of surrounding soft tissues** (median, range)			
Noncontrast phase	36.2 (10.0, 62.0)	34.3 (-86.8, 120.0)	0.379
Portovenous phase	52.6 (16.0, 110.0)	44.8 (-43.1, 182.2)	*0.028*
Difference between HU of appendicolith and surrounding soft tissues in noncontrast phase	58.6 (-44.6, 91.0)	98.9 (-112, 1498.4)	*<0.001*
Difference between HU of appendicolith and surrounding soft tissues in portovenous phase	34.5 (-98.7, 82.7)	83.5 (-180.5, 1479.6)	*<0.001*

HU = Hounsfield Unit

Supplementary Material 3: Computed tomographic characteristics of appendicitis with appendicoliths not shown on portovenous phase (n=24)

Case No.	Final diagnosis	CT diagnosis	Wall enhancement defect	Moderate-severe fat stranding	Fluid collection	Extraluminal air
24	Uncomplicated	Uncomplicated	Yes	Yes	Yes	Yes
27	Complicated	Complicated	Yes	Yes	Yes	Yes
51	Complicated	Complicated	Yes	Yes	Yes	Yes
55	Complicated	Complicated	Yes	Yes	Yes	Yes
99	Complicated	Uncomplicated	No	Yes	No	No
102	Complicated	Complicated	Yes	Yes	No	No
115	Complicated	Complicated	Yes	Yes	No	No
119	Complicated	Complicated	Yes	Yes	Yes	Yes
131	Complicated	Complicated	Yes	Yes	No	Yes
149	Complicated	Uncomplicated	No	Yes	No	No
168	Complicated	Complicated	Yes	Yes	Yes	Yes
169	Uncomplicated	Complicated	Yes	Yes	No	No
174	Uncomplicated	Uncomplicated	No	No	No	No
175	Complicated	Complicated	Yes	Yes	Yes	Yes
210	Uncomplicated	Complicated	Yes	Yes	No	No
214	Complicated	Complicated	No	Yes	No	No
219	Complicated	Complicated	Yes	Yes	Yes	Yes
223	Uncomplicated	Uncomplicated	No	Yes	No	No
226	Complicated	Complicated	Yes	Yes	No	No
232	Complicated	Complicated	Yes	Yes	Yes	No
241	Uncomplicated	Complicated	Yes	Yes	Yes	Yes
246	Complicated	Complicated	Yes	Yes	Yes	Yes
250	Uncomplicated	Uncomplicated	No	No	No	No
290	Uncomplicated	Uncomplicated	No	Yes	No	No
